# mwHIT: accelerated and accurate histone modification imputation using multi-scale window attention

**DOI:** 10.3389/fgene.2026.1896629

**Published:** 2026-07-13

**Authors:** Zhaoxi Zhang, Lijuan Jia, Xiaoya Fan, Zengyou He, Juan Wang, Zhong Wang

**Affiliations:** 1 School of Software, Dalian University of Technology, Dalian, China; 2 School of Computer Science, Inner Mongolia University, Hohhot, China; 3 Beijing Key Laboratory of Topological Statistics and Applications for Complex Systems, Beijing Institute of Mathematical Sciences and Applications, Beijing, China

**Keywords:** histone modification, run-on sequencing, DNA Sequence, transformer, multi-scale window attention

## Abstract

Histone modifications regulate chromatin organization and gene activity, and abnormal modification patterns are closely associated with disease-related transcriptional dysregulation. Although ChIP-seq provides accurate measurements of histone modification profiles, genome-wide experimental profiling remains costly and time-consuming. Here, we propose mwHIT, a multi-scale window attention-based Histone Modification Imputation Transformer that predicts histone modification signals from run-on sequencing (RO-seq) and DNA sequence features. mwHIT captures both local and long-range regulatory information while reducing the computational cost of transformer-based inference. Across cell lines and histone marks, mwHIT achieved an average Pearson correlation of 0.7086 in matched method comparisons, outperforming dHIT and Enformer by 13.7% and 33.4%, respectively. On the GM12878 benchmark, mwHIT reduced average mean squared error (MSE) by 69.7% relative to dHIT and 34.6% relative to Enformer. Compared with a full-attention transformer baseline, the multi-scale window attention design reduced runtime from 0.05 h to 0.03 h while maintaining comparable or slightly higher predictive accuracy. mwHIT also recovered histone modification patterns in promoters, enhancers, gene bodies, and regions centered on transcription start sites (TSSs), and highlighted disease-associated regulatory signals near the IL2RA locus. These results demonstrate that mwHIT can support the discovery of disease-associated regulatory markers and provide computational evidence for prioritizing candidate regulatory regions and potential regulatory targets. mwHIT’s data processing methods and model code are freely available in the mwHIT GitHub repository at https://github.com/zhichunlizzx/mwHIT.

## 1 Introduction

Profiling histone modifications is important for biological and medical research. Aberrant histone modifications can alter the expression of key genes and contribute to diseases, including cancer (Jones and Baylin, 2002). From a medical perspective, such aberrant modification patterns may provide candidate biomarkers or therapeutic targets (Dawson and Kouzarides, 2012). At present, the high experimental cost and resource demands of histone modification profiling have limited the discovery and clinical validation of relevant targets. Incorrect targets caused by the lack of histone regulatory information can make drugs ineffective or even cause adverse reactions in patients (Yin et al., 2020; Macarron et al., 2011; Bowes et al., 2012; Gashaw et al., 2011). Therefore, generating reliable histone modification profiles in disease samples is essential for prioritizing clinically relevant biomarkers and therapeutic targets.

Chromatin immunoprecipitation sequencing (ChIP-seq) uses specific antibodies to enrich histone modifications and their bound DNA fragments. High-throughput sequencing is then used to determine the genomic location and abundance of these modifications (Park, 2009; Furey, 2012). It is currently the most accurate and reliable method for measuring histone modification profiles (Barski et al., 2007; Johnson et al., 2007). However, ChIP-seq requires substantial labor, time, and financial resources. Currently available ChIP-seq datasets (Jou et al., 2019; Barrett et al., 2013) remain insufficient to support broad biomedical applications (Furey, 2012; Landt et al., 2012). Computational methods can partially overcome the limitations of experimental approaches (Zhang Z. et al., 2022). Computational methods usually use classification or regression algorithms to analyze the DNA data or epigenomic data provided by researchers to predict the target experimental signal. In the past, statistical machine learning algorithms such as random forests, naive Bayes, and support vector machines were often used as classification algorithms (Zhang Z. et al., 2022; Libbrecht and Noble, 2015; Kohavi and John, 1997; Yao, 1997; Kulmanov and Hoehndorf, 2021; Zou, 2019). However, these algorithms often lack sufficient predictive accuracy, limiting the widespread use of computational methods for biological signal prediction.

Advances in deep learning (LeCun et al., 2015) offer new opportunities to address this problem. Deep learning has been applied to many genomic and transcriptomic tasks, including epigenomic information (Yin et al., 2019; Benveniste et al., 2014; Quang and Xie, 2019; Žiga et al., 2021; Kelley et al., 2018; Quang and Xie, 2016), gene mutations (Zhou and Troyanskaya, 2015; Zhou et al., 2018), gene expression levels (Singh et al., 2017; Karollus et al., 2023; Karbalayghareh et al., 2022), 3D chromatin architecture (Schreiber et al., 2018; Fudenberg et al., 2020), and enhancer activity prediction (Sethi et al., 2020). Compared with traditional biological experimental methods, deep learning has the advantages of speed, low cost, and broad applicability. Although deep-learning-based predictions are generally less accurate than ChIP-seq measurements, they hold great potential for time-sensitive biomedical studies and large-scale functional genomic analyses. Most of these methods use DNA sequence or chromatin accessibility information (Sethi et al., 2020) as input to deep learning algorithms. However, these inputs may lose information related to cell-type specificity or transcriptional directionality.

Run-on sequencing (RO-seq), represented by PRO-seq, GRO-seq, and ChRO-seq, provides strand-resolved maps of nascent transcription across the genome (Chu et al., 2018). RO-seq signals are strongly correlated with histone modifications associated with transcriptional activation and are therefore used in various biological studies. Danko et al. (Danko et al., 2015) used an SVM to develop a peak detection model dREG for RO-seq data. Subsequently, Wang et al. (Wang et al., 2019) improved dREG using the CUDA framework, allowing dREG to run on GPUs and speeding up the computation. Recently, Wang et al. (Wang Z. et al., 2022) proposed a machine learning method, dHIT. dHIT used RO-seq data and support vector regression to predict the levels of 10 histone modifications and achieved strong predictive performance. However, dHIT still has several limitations. First, SVR, as a type of statistical machine learning, cannot effectively model long-range transcriptional regulatory dependencies. Second, dHIT only uses RO-seq data, and its performance remains limited when predicting histone modifications that play a role in transcriptional repression. Third, the computational efficiency of dHIT is relatively poor. It takes several hours to complete the prediction of chromosome 22. The strong performance of transformer models (Ashish et al., 2017) in natural language processing (Devlin et al., 2019; Brown et al., 2020) and machine vision (Dosovitskiy et al., 2020) inspired us to improve the dHIT model. Transformer models can address these limitations of dHIT owing to their ability to model long-range dependencies. Furthermore, transformer architectures based on the shifted-window attention mechanism (Liu et al., 2021) have effectively reduced the computational complexity of the model. These properties make transformer architectures well suited for genomic data.

**FIGURE 1:**
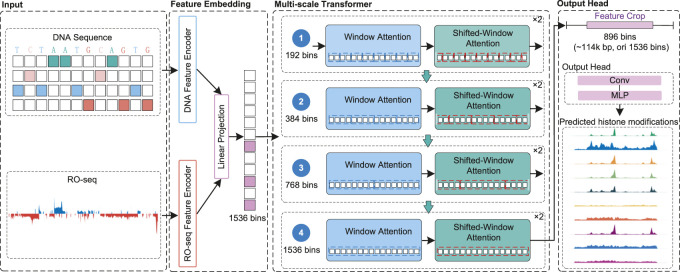
Model architecture of mwHIT. The model input consists of the one-hot encoding of the DNA sequence and three-channel RO-seq features. The model includes: (1) Feature encoding, including feature embedding blocks for RO-seq and DNA sequence inputs, implemented using convolutional neural network blocks. (2) Multi-scale transformer block. It contains eight transformer modules consisting of window-attention and shifted-window-attention layers. (3) Output block. It crops the transformer output to the target length. Next, convolutional layers and fully connected layers are used to predict the signal levels of 10 histone modifications.

Motivated by these challenges, we developed mwHIT (Figure 1), a Histone Imputation Transformer that extends dHIT in both input representation and model architecture. Unlike dHIT, which relies on support vector regression with RO-seq features, mwHIT integrates RO-seq and DNA sequence information in an end-to-end deep learning framework and uses a multi-scale window attention mechanism to capture regulatory dependencies at different genomic scales. This design is intended to retain the long-range modeling capacity of transformer architectures while avoiding the high computational cost of full attention during genome-wide inference. We systematically evaluate mwHIT on held-out chromosomes, cross-cell-line prediction tasks, comparisons with existing imputation methods, and a full-attention transformer baseline. We further examine whether the predicted histone modification profiles recover known regulatory patterns around TSSs and functionally annotated genomic regions, and whether they can highlight disease-associated regulatory loci. This comprehensive evaluation illustrates how mwHIT can provide regulatory evidence for prioritizing disease markers and candidate regulatory targets.

## 2 Materials and methods

### 2.1 Datasets

**FIGURE 2:**
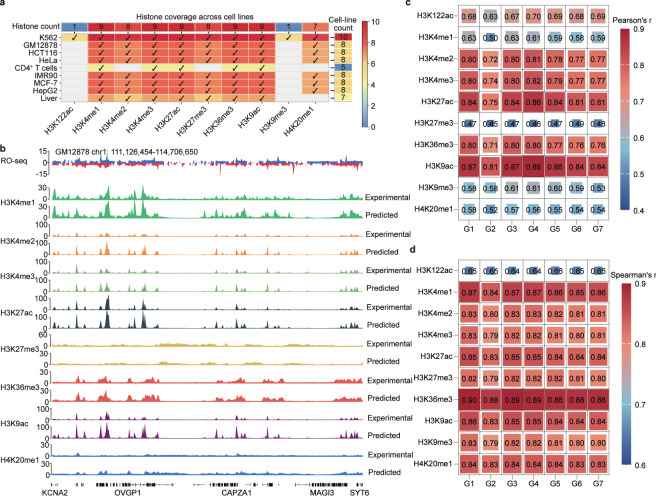
Dataset coverage and held-out chromosome prediction performance of mwHIT. **(a)** Overview of the training and evaluation datasets, including K562 G1–G7 samples used for model training and held-out-chromosome testing, together with independent cell lines and tissues used for generalization evaluation. **(b)** Genome-browser visualization comparing experimentally measured and mwHIT-predicted histone modification profiles in the GM12878 cell line. **(c)** Pearson correlation of predictions for individual histone marks on chromosome 22 using K562 G1–G7 samples. **(d)** Spearman correlation of predictions for individual histone marks on chromosome 22 using K562 G1–G7 samples.

To develop and assess the model, we collected datasets covering eight human cell lines, namely, K562, GM12878, HCT116, HeLa-S3, ${CD4}^{+}$ T cells, IMR90, MCF-7, and HepG2, and additionally incorporated mouse liver data. The predicted histone modifications were H3K122ac, H3K4me1, H3K4me2, H3K4me3, H3K27ac, H3K36me3, and H3K9ac, which indicate transcriptional activation (Barski et al., 2007; Koch et al., 2007; Heintzman et al., 2007), and H3K27me3, H3K9me3, and H4K20me1, which indicate transcriptional repression (Mikkelsen et al., 2007). Due to the lack of corresponding ChIP-seq histone modification data, we evaluated eight histone modifications in cell lines other than K562 (Figure 2a). Existing deep learning prediction algorithms often only use data from a single sample in a single cell line for model training, which limits the generalization ability of the model in cross-cell-line prediction tasks (Yin et al., 2019; Žiga et al., 2021; Wang et al., 2019). Here, we used seven RO-seq samples (G1-G7) from the K562 cell line to train the model. A held-out RO-seq sample (G8) was used for genome-wide evaluation in K562. In this study, the reference genomes for human and mouse data were hg19 and mm10, respectively. All RO-seq and histone modification ChIP-seq data sources are provided in the mwHIT GitHub repository at https://github.com/zhichunlizzx/mwHIT.

The data were partitioned into training, validation, and test sets. The training and validation sets included all genomic regions of the K562 cell line except chromosome 22. When defining training and validation samples, we chose genomic regions enriched for RO-seq or histone modification signals as positive samples. These regions were defined based on peak-calling results of various sequencing datasets. Regions other than positive samples were defined as negative samples. These samples were split into training and validation sets at an 80:20 ratio. The test set consisted of chromosome 22 from the K562 cell line and the genome-wide regions from the other cell lines. Finally, our training and validation sets contained more than 160,000 samples, and the whole-genome test set contained more than 26,000 non-overlapping samples. Each sample corresponded to a genomic region of 114 kb in length.

### 2.2 Feature extraction

The inputs to mwHIT include DNA sequence and RO-seq signals. We used a four-dimensional one-hot encoding (Alipanahi et al., 2015) to represent the DNA sequence (N = [0, 0, 0, 0], A = [1, 0, 0, 0], G = [0, 0, 1, 0], C = [0, 1, 0, 0], T = [0, 0, 0, 1]). RO-seq signals were represented using three channels, which is the positive-strand signal, negative-strand signal, and the sum of the positive- and negative-strand signals. The ground-truth signal was obtained from the bigWig file for the corresponding histone modification. We set the resolution of the model to 128 bp, so the ground-truth histone modification signal for each sample contains 896 values. Each value in the ground-truth represents the average of all signals in the corresponding genomic region. *NaN* and Inf values in RO-seq and histone modification ChIP-seq data were replaced with $10^{-9}$ . To ensure that the model could observe sufficient upstream and downstream information when processing the data at either end of each sample, we extended each sample by 41 kb at both ends. In this way, the sample length reached 197 kb, and these extended regions were clipped before the output layer. As a result, the dimensions of the data used in this paper are: [197k, 4] (DNA), [197k, 3] (RO-seq), [896, 10] (histone modification).

### 2.3 Multi-scale window attention

Transformer models have shown a strong ability to model long-range regulatory dependencies in various epigenetic information prediction tasks (Žiga et al., 2021). However, in multi-head attention (MHA), the computational complexity increases quadratically with sequence length. For sequences with length *L*, the computational complexity of MHA is shown in Equation 1:

$$\Omega \left(\text{MHA}\right)=4LC^{2}+2L^{2}C$$ (1)

where *C* denotes the feature dimension at each genomic position in a sequence of length *L*. The computational complexity of the window multi-head attention (W-MHA) mechanism is shown in Equation 2:

$$\Omega \left(\text{W}-\text{MHA}\right)=4LC^{2}+2WLC$$ (2)

Where, *W* denotes the window size. When *W* is a fixed value, the second term in the W-MHA complexity is linear in *L*. Therefore, using W-MHA can reduce the computational cost of the model and accelerate model inference. In this study, we adapted W-MHA to preserve the sequence length throughout the model. Therefore, we did not use downsampling operations to merge the input features. The window size was kept fixed within each attention stage. We used window sizes ranging from 192 to 1536 to extract regulatory features across multiple genomic scales. By using multi-scale W-MHA, mwHIT achieved an approximately 1.7-fold inference speedup compared with the full-attention transformer baseline.

### 2.4 Network design

mwHIT used a transformer architecture based on a multi-scale window attention mechanism as the backbone network. The architecture of mwHIT is shown in Figure 1. mwHIT mainly comprises three parts: feature embedding block, transformer block, and output block. The feature embedding block consists of seven repeated convolutional layers and max-pooling layers. For the convolutional operation, we used a kernel size of 15 and a stride of 1. The pool size of the max-pooling layer is 2. The input is compressed into a vector of length 1536 after the convolution block, and each point in the vector represents genomic features within a 128-bp bin. During the convolution process, the number of channels for each point gradually increases from 3 or 4 input channels to 768 channels. We used two feature embedding blocks with the same structure to transform DNA sequence data and RO-seq data into tensors with shape (1536, 768), respectively. Then, the results of the two feature extraction blocks were combined using learnable weights and fed into the transformer module. The transformer block includes 16 transformer layers, each of which consists of a multi-head attention module and a multi-layer perceptron module. The transformer block is divided into eight stages, each of which contains a transformer layer and a shifted-window transformer layer. The number of heads used in these eight stages is 16, 16, 16, 16, 8, 8, 8, and 8, respectively. The corresponding window sizes are 192, 192, 384, 384, 768, 768, 1536, and 1536. The input and output dimensions of the transformer block remain unchanged. Its function is to learn local and long-range interactions from the data to predict the histone modification levels across the whole genome. The output module contains a clipping module, a convolutional layer, and a fully connected layer. Its purpose is to map the learned regulatory representations to 10 histone modification signals. The clipping module removes the extended flanking regions when extracting features. In this study, the regularization methods used included BatchNorm, LayerNorm, DropPath, and Dropout (Zhang X. et al., 2022; Huang et al., 2016; Wang T. et al., 2022). Residual connections (He et al., 2016) were extensively used to mitigate vanishing gradients.

### 2.5 Model training

To speed up training and evaluation, we stored the input features and ground-truth signals in TFRecord files. During model training and evaluation, these data were loaded by GPU data iterators. In each epoch, we randomly selected 10,000 samples for model training. The model was optimized using Adam, with MSE adopted as the training objective. mwHIT used a learning-rate decay strategy to prevent the model from overfitting. We set the initial learning rate to $10^{-4}$ . After each epoch, the learning rate was reduced to one-third of the previous epoch’s learning rate. All training and evaluation experiments were performed using 32 GB NVIDIA Tesla V100 GPUs. By using multi-GPU training, the training and prediction time could be further shortened.

### 2.6 Evaluation

In this study, we used a variety of evaluation metrics to evaluate the predictive performance of mwHIT and compare it with other histone modification prediction models, including Pearson correlation, Spearman correlation, and six MSE-based metrics (Zhang et al., 2023; Schreiber et al., 2020; Schreiber et al., 2023). The definitions of the six MSE metrics are shown in Table 1.

**TABLE 1:** Six MSE-based metrics used to evaluate epigenomic signal prediction.

Abbreviation	Description
mseGlobal	The genome-wide MSE
mseGene	The MSE in the protein-coding genes
mseProm	The MSE in the promoter regions
mseEnh	The MSE in the enhancer regions
mseObs	The MSE at the top 1% of genomic positions ranked by experimental signal
mseImp	The MSE at the top 1% of genomic positions ranked by predicted signal

We also used ROC curves (Fawcett, 2006), PR curves, and their corresponding AUC and AUPR values to evaluate mwHIT. When one method’s ROC curve lies above another across most thresholds, this indicates superior discrimination performance. Method performance can also be summarized using AUC and AUPR values.

## 3 Results

We evaluated mwHIT from complementary perspectives, including genome-browser visualization, held-out chromosome prediction, cross-cell-line generalization, and comparisons with existing methods. Unless otherwise specified, genome-wide correlation analyses were performed at 1 kb resolution by averaging adjacent 128 bp predictions and experimental signals.

### 3.1 mwHIT accurately predicts multiple types of histone modifications

We first examined whether mwHIT could reproduce genome-wide histone modification profiles from RO-seq and DNA sequence inputs. The study design and available histone modification datasets are summarized in Figure 2a. In the GM12878 cell line, the predicted profiles closely followed the experimentally measured ChIP-seq signals across representative genomic regions, including both sharp promoter-associated marks and broader gene-body or repressive marks (Figure 2b). Quantitatively, mwHIT achieved an average Pearson correlation of 0.7805 ± 0.1058 and an average Spearman correlation of 0.6053 ± 0.0545 across the available GM12878 histone modification assays. This visual and quantitative agreement indicates that mwHIT captures the major signal patterns of multiple histone modifications rather than only predicting isolated peak positions.

We next evaluated the model’s predictive accuracy using the held-out chromosome in K562. The seven K562 RO-seq replicates used for model training (G1–G7) were evaluated on chromosome 22, which was excluded from training and validation. Across these held-out evaluations, mwHIT achieved consistently high Pearson and Spearman correlations for most histone modification types (Figure 2c,d), with mean values of 0.6887 ± 0.1286 and 0.8166 ± 0.0619, respectively, across the G1–G7 held-out evaluations. Prediction accuracy was especially strong for transcriptionally activating marks, with mean Pearson correlations of 0.8535 ± 0.0261 for H3K9ac, 0.8214 ± 0.0359 for H3K27ac, 0.7843 ± 0.0262 for H3K4me3, and 0.7799 ± 0.0298 for H3K4me2. These results show that the model generalizes to genomic regions not seen during training. Some repressive marks showed lower Pearson correlations, consistent with their weaker direct coupling to transcriptional activity.

**FIGURE 3:**
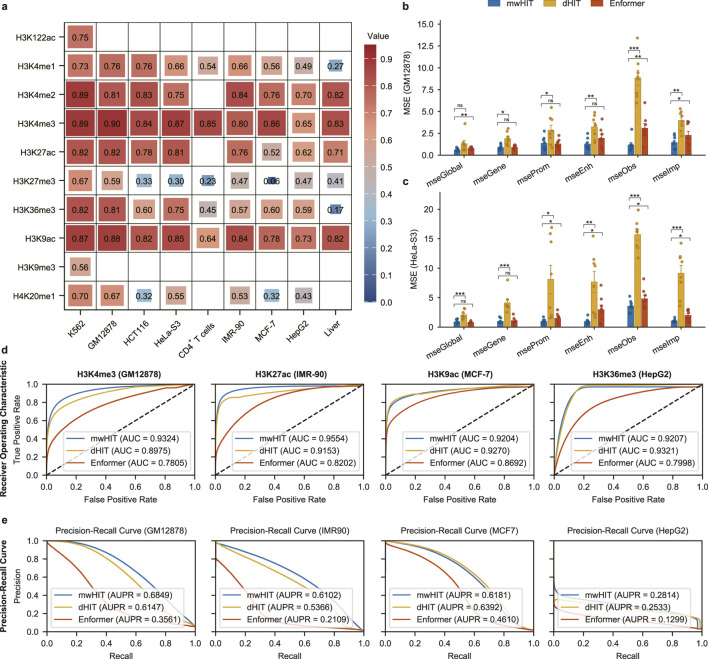
Cross-cell-line evaluation and comparison with existing methods. **(a)** Pearson correlation between experimental and predicted histone modification profiles across cell lines and tissues at 1 kb resolution, showing the generalization performance of mwHIT beyond the training cell line. **(b,c)** Comparison of MSE-based metrics between mwHIT and existing methods in GM12878 **(b)** and HeLa-S3 **(c)** across representative histone modification types. Bar plots indicate the mean MSE (bar height) with standard error of the mean (error bars), with individual data points overlaid on each bar. Statistical significance was assessed using paired two-sided Student’s t-tests without adjustment for multiple comparisons (* P<0.05 , ** P<0.01 , *** P<0.001 ). **(d,e)** ROC and precision-recall curve comparison for representative histone modification–cell line pairs: H3K4me3 in GM12878, H3K27ac in IMR-90, H3K9ac in MCF-7, and H3K36me3 in HepG2. These curves evaluate the ability of different methods to distinguish modified from unmodified genomic regions.

A key criterion for evaluating a histone modification prediction model is its ability to generalize across different cell lines. We evaluated mwHIT using histone modification data from eight human cell lines and mouse liver tissue. The cross-cell-line evaluation results are summarized in Figure 3a. mwHIT achieved high correlations for most histone modification types across diverse cellular contexts, with an average Pearson correlation of 0.7086 ± 0.1808 across the cell-line and histone-mark combinations used for method comparison. Individual active marks reached correlations above 0.8 in multiple cell lines; for H3K4me3, Pearson correlations reached 0.9048 in GM12878, 0.8729 in HeLa-S3, and 0.8495 in ${CD4}^{+}$ T cells. These results support the ability of mwHIT to generalize from K562 to other cell lines and tissues. Differences among cell lines and histone modification types remained, especially for repressive marks, suggesting that cell-type-specific chromatin states and transcription-independent regulatory information still contribute to prediction difficulty.

### 3.2 mwHIT outperforms existing histone imputation methods

**FIGURE 4:**
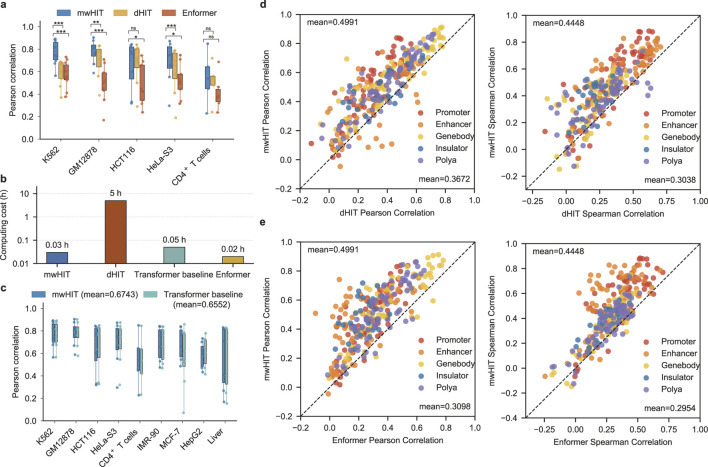
Comparison of mwHIT with existing methods and transformer baselines. **(a)** Comparison of cross-cell-line Pearson correlations among mwHIT, dHIT, and Enformer. Each data point represents the Pearson correlation for a distinct histone modification within a cell line. Box plots show the median (center line), interquartile range (box bounds), and whiskers extending to 1.5 × IQR. Statistical significance was assessed using paired two-sided Student’s t-tests without adjustment for multiple comparisons (* P<0.05 , ** P<0.01 , *** P<0.001 ). **(b)** Computational cost comparison for chromosome 22 histone modification prediction; dHIT was evaluated on a TITAN XP GPU, whereas the other methods were evaluated on Tesla V100 GPUs. **(c)** Pearson correlation comparison between the multi-scale window transformer and the full-attention transformer baseline. **(d,e)** Pearson and Spearman correlation comparisons in functionally annotated genomic regions.

We next compared mwHIT with existing histone modification prediction methods, including dHIT (Wang Z. et al., 2022) and the transformer-based Enformer model (Žiga et al., 2021). In cross-cell-line Pearson correlation comparisons, mwHIT generally achieved comparable or higher accuracy across the evaluated cell lines and histone modification types (Figure 4a). Across matched comparisons, mwHIT obtained an average Pearson correlation of 0.7086 ± 0.1808, compared with 0.6234 ± 0.1706 for dHIT and 0.5312 ± 0.1396 for Enformer. This corresponds to average improvements of 13.7% (0.0852) and 33.4% (0.1773) over dHIT and Enformer, respectively. The advantage was most apparent for several transcription-associated active marks, where RO-seq provides direct information about ongoing transcriptional activity. For example, in GM12878, mwHIT improved H3K4me1 prediction from 0.6700 to 0.7636 relative to dHIT, a 14.0% increase in Pearson correlation, and from 0.3500 to 0.7636 relative to Enformer, a 118.2% increase. The comparison also showed that several repressive marks remained challenging. Unlike active marks that often exhibit sharp and transcription-associated peaks, repressive marks such as H3K27me3 and H3K9me3 tend to form broad and continuous domains with less clearly defined boundaries, making their cell-type-specific signal patterns more difficult to recover.

We further evaluated prediction errors using six MSE-based metrics that quantify genome-wide accuracy and performance in functionally relevant genomic regions. On the GM12878 benchmark, mwHIT obtained lower MSE values than the comparison methods for most histone modifications and genomic-region metrics (Figure 3b,c), with an average MSE of 1.1171 ± 0.6505 compared with 3.6879 ± 2.8427 for dHIT and 1.7075 ± 1.2379 for Enformer. These values correspond to MSE reductions of 69.7% relative to dHIT and 34.6% relative to Enformer. In absolute terms, the average MSE decreased by 2.5708 relative to dHIT and by 0.5904 relative to Enformer. This suggests that the improvement is reflected not only in correlation but also in signal amplitude. Peak-level evaluation showed a similar trend. For representative activation- and repression-associated marks, the ROC and precision-recall curves showed that mwHIT achieved competitive discrimination between modified and unmodified regions compared with dHIT and Enformer (Figure 3d,e).

### 3.3 Multi-scale window attention improves efficiency while preserving accuracy

To assess the contribution of the multi-scale window attention design, we compared mwHIT with a full-attention transformer baseline that uses the same feature embedding and output modules but does not use window attention. mwHIT, the window-attention model, completed the chromosome 22 benchmark prediction in 0.03 h, whereas the full-attention transformer baseline required 0.05 h (Figure 4b). This corresponds to a 40.0% reduction in runtime, or an absolute decrease of 0.02 h, which is equivalent to an approximately 1.7-fold speedup. In the same benchmark, dHIT required 5 h and Enformer required 0.02 h (Figure 4b). Thus, mwHIT was substantially faster than dHIT and the full-attention transformer baseline, while maintaining stronger predictive performance than Enformer in matched comparisons.

The speedup did not come at the expense of prediction accuracy. Across the cell-line and histone modification comparisons in the transformer benchmark, the multi-scale window transformer achieved a mean Pearson correlation of 0.6743 ± 0.1810, compared with 0.6552 ± 0.2039 for the full-attention transformer baseline (Figure 4c), representing a 2.9% improvement and an absolute Pearson increase of 0.0191. The two models showed broadly similar performance across most tasks, and the full-attention transformer baseline performed slightly better than mwHIT in a few settings. However, the window-attention model achieved clear gains in several cross-cell-line predictions, including MCF-7 H3K27me3, where Pearson correlation increased from 0.0710 to 0.4858. These results suggest that multi-scale window attention can retain, and in some cases improve, predictive performance while making genome-wide inference faster.

### 3.4 mwHIT identifies biologically meaningful genomic regions

We further asked whether mwHIT predictions preserve signals in functionally meaningful genomic regions rather than only improving genome-wide summary metrics. We evaluated predictions in promoters, enhancers, gene bodies, insulators, and polyadenylation-associated regions. Promoter and enhancer annotations were derived from dREG regulatory element annotations (Wang et al., 2019). Gene body annotations were obtained from NCBI genome annotations for hg19 (GCF_000001405.25) and mm10 (GCF_000001635.20). Poly(A)-associated regions were defined as the 200-bp regions downstream of gene ends, and TSS-centered regions were defined as 200-bp windows centered on gene starts. Insulator regions were obtained from annotations provided by Ernst et al. (Ernst and Kellis, 2010; Ernst et al., 2011). Across these regions, mwHIT showed higher agreement with experimental signals than the comparison methods (Figure 4d,e). The mean Pearson correlation of mwHIT was 0.4991 ± 0.2075, compared with 0.3672 ± 0.2006 for dHIT and 0.3098 ± 0.1838 for Enformer, corresponding to improvements of 35.9% and 61.1%, respectively. In absolute terms, Pearson correlation increased by 0.1319 relative to dHIT and by 0.1893 relative to Enformer. The same trend was observed for rank-based agreement, with mwHIT achieving a mean Spearman correlation of 0.4448 ± 0.2125 compared with 0.3038 ± 0.1894 for dHIT and 0.2954 ± 0.1612 for Enformer. These results indicate that mwHIT more accurately recovers histone modification patterns in genomic regions with established regulatory functions.

**FIGURE 5:**
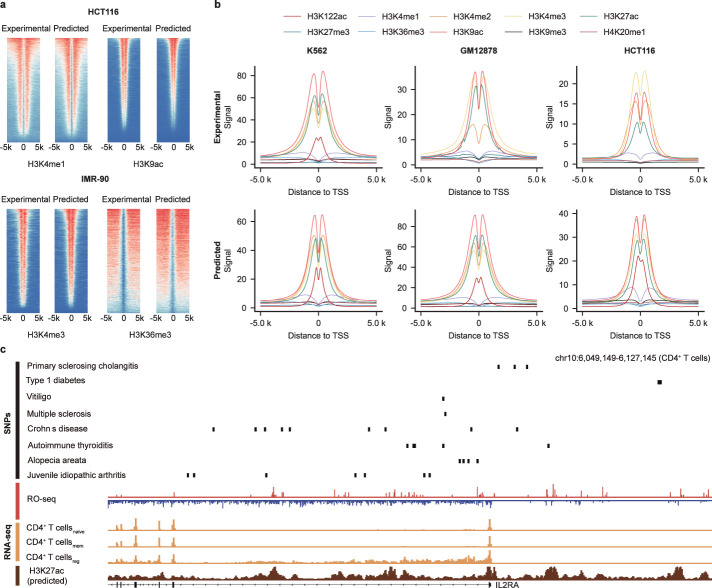
mwHIT predictions recapitulate biologically meaningful regulatory patterns. **(a)** Heatmap comparison of experimental and predicted histone modification profiles around TSSs in representative cell lines, showing whether predicted signals preserve promoter-proximal enrichment patterns. **(b)** Aggregate experimental and predicted histone modification profiles centered on TSSs, summarizing the average signal distribution upstream and downstream of transcription start sites. **(c)** Predicted H3K27ac signals, RNA-seq, RO-seq, and autoimmune disease-associated SNPs near the IL2RA locus in ${CD4}^{+}$ T cells, illustrating the potential use of mwHIT predictions for prioritizing disease-associated regulatory regions.

The biological consistency of the predicted profiles was also evident around TSSs. In representative cell lines, predicted histone modification profiles recapitulated the experimentally observed enrichment patterns near TSSs (Figure 5a). Aggregate profiles further showed that active marks, including H3K4me3, H3K9ac, and H3K27ac, formed characteristic peaks around the TSS in both experimental and predicted signals (Figure 5b). These patterns suggest that mwHIT learns regulatory signal organization associated with promoter-proximal transcriptional activity.

Finally, we examined a disease-relevant regulatory locus. At the IL2RA locus in ${CD4}^{+}$ T cells, predicted H3K27ac signals overlapped multiple discrete regulatory subregions near clusters of autoimmune disease-associated single-nucleotide polymorphisms (SNPs) (Figure 5c). Prior genetic and epigenetic fine-mapping work has shown that autoimmune disease variants are enriched in active regulatory elements, including enhancer-rich regions marked by H3K27ac (Farh et al., 2015). The IL2RA example suggests that mwHIT predictions can help prioritize candidate regulatory regions where disease variants may affect cell-type-specific enhancer activity.

## 4 Conclusion

In this study, we developed a genome-wide histone modification imputation workflow based on RO-seq and DNA sequence features and evaluated it across multiple computational and biological criteria. We tested mwHIT on held-out chromosomes and cross-cell-line datasets, compared it with existing imputation methods and a full-attention transformer baseline, and examined whether its predictions recapitulate signals in biologically meaningful regulatory regions. These analyses covered both global prediction accuracy and functional genomic contexts, including TSS-centered patterns, regulatory-region-specific performance, and a disease-associated IL2RA locus.

Overall, mwHIT showed advantages in three main aspects. First, in terms of prediction accuracy, mwHIT achieved strong agreement with experimental histone modification profiles and improved both Pearson correlation and MSE metrics compared with dHIT and Enformer in matched evaluations. This advantage was particularly evident for transcriptionally active marks such as H3K9ac, H3K27ac, and H3K4me3. Second, mwHIT showed robust cross-cell-line generalization, maintaining high predictive performance across independent cell lines and tissues beyond the K562 training data. Third, mwHIT improved computational efficiency through its multi-scale window attention design. Compared with the full-attention transformer baseline, mwHIT reduced the benchmark inference runtime from 0.05 h to 0.03 h while preserving comparable or slightly higher accuracy. In addition, mwHIT retained meaningful signal patterns around TSSs and functional genomic regions, suggesting that the model learns regulatory structure rather than only optimizing global similarity metrics.

Despite these advantages, mwHIT still has limitations. Prediction remains more challenging for some repressive marks and cell-type-specific chromatin states, likely because RO-seq and DNA sequence do not capture all regulatory information needed for these contexts. Future work could incorporate additional epigenomic modalities, improve feature fusion for transcriptionally repressed regions, and evaluate mwHIT on broader disease and tissue datasets. With these extensions, mwHIT may provide a more scalable computational framework for histone modification imputation and regulatory-region prioritization in functional genomics studies.

## Data Availability

The data presented in the study are deposited in the mwHIT GitHub repository, available at https://github.com/zhichunlizzx/mwHIT. The datasets analyzed in this study were obtained from publicly available repositories, and the corresponding accession IDs, download information, and source code are provided in this repository.
